# *Cryptosporidium* Lactate Dehydrogenase Is Associated with the Parasitophorous Vacuole Membrane and Is a Potential Target for Developing Therapeutics

**DOI:** 10.1371/journal.ppat.1005250

**Published:** 2015-11-12

**Authors:** Haili Zhang, Fengguang Guo, Guan Zhu

**Affiliations:** Department of Veterinary Pathobiology, College of Veterinary Medicine & Biomedical Sciences, Texas A&M University, College Station, Texas, United States of America; University of Wisconsin Medical School, UNITED STATES

## Abstract

The apicomplexan, *Cryptosporidium parvum*, possesses a bacterial-type lactate dehydrogenase (CpLDH). This is considered to be an essential enzyme, as this parasite lacks the Krebs cycle and cytochrome-based respiration, and mainly–if not solely, relies on glycolysis to produce ATP. Here, we provide evidence that in extracellular parasites (e.g., sporozoites and merozoites), CpLDH is localized in the cytosol. However, it becomes associated with the parasitophorous vacuole membrane (PVM) during the intracellular developmental stages, suggesting involvement of the PVM in parasite energy metabolism. We characterized the biochemical features of CpLDH and observed that, at lower micromolar levels, the LDH inhibitors gossypol and FX11 could inhibit both CpLDH activity (*K*_i_ = 14.8 μM and 55.6 μM, respectively), as well as parasite growth *in vitro* (IC_50_ = 11.8 μM and 39.5 μM, respectively). These observations not only reveal a new function for the poorly understood PVM structure in hosting the intracellular development of *C*. *parvum*, but also suggest LDH as a potential target for developing therapeutics against this opportunistic pathogen, for which fully effective treatments are not yet available.

## Author Summary

Cryptosporidians are unique among the apicomplexans in regards to their parasitic life style (e.g., they are intracellular, but undergo extracytoplasmic development within a host membrane-derived structure termed parasitophorous vacuole membrane, PVM) and their metabolism (e.g., they are incapable of *de novo* nutrient synthesis and rely on glycolysis for the synthesis of ATP). We discovered that the *Cryptosporidium parvum* bacterial-type L-lactate dehydrogenase (CpLDH) enzyme is cytosolic during the parasite’s motile, extracellular, stages (sporozoites and merozoites), but becomes associated with the PVM during intracellular development, indicating the involvement of the PVM in lactate fermentation. We also observed that micromolar concentrations of the LDH inhibitors gossypol and FX11 inhibit both CpLDH activity and the growth of *C*. *parvum in vitro*, suggesting that CpLDH is a potential target for the development of anti-cryptosporidial therapeutics.

## Introduction

*Cryptosporidium parvum* is a gastrointestinal parasite that can cause moderate to severe diarrhea in children and adults, and deadly opportunistic infection in AIDS patients [1, 2]. In addition, because *Cryptosporidium* oocysts are resistant to chemical stresses, such as chlorine treatment, it also frequently causes water-borne outbreaks around the world [3, 4]. Current treatment options for cryptosporidiosis are limited [1, 5]. In the United States, only nitazoxanide is approved by the Federal Drug Administration (FDA) to treat cryptosporidial infections in immunocompetent individuals, but this is not approved for immunocompromised patients [6–8].

*Cryptosporidium* shares many biological features with other apicomplexans. They all undergo similar stages of life cycle development, including the invasion of sporozoites into host cells after excystation from oocysts, followed by varied cycles of merogony to form merozoites, gametogenesis to form micro- and macro-gametes, fertilization, and oocyst formation. The sporozoites and meorzoites also contain an apical complex consisting of a number of unique cytoskeletal structures and secretory organelles, such as rhoptries and micronemes. During the intracellular development, *Cryptosporidium* and most other apicomplexans reside within a vacuole termed parasitophorous vacuole, although some escape from the vacuole shortly after invasion (e.g., *Theileria* and *Babesia*). The vacuole is formed during the parasite invasion and defined by a host cell-derived membrane structure termed parasitophorous vacuole membrane (PVM). However, *Cryptosporidium* also differs from other apicomplexans in that these parasites lack both an apicoplast and a typical mitochondrion, and are incapable of the *de novo* synthesis of amino acids, fatty acids, and nucleosides. Additionally, they undergo a unique intracellular, but extracytoplasmic development, in which the PVM faces the extracellular environment, rather than the host cell cytosol [9–11].

Energy metabolism in some members of the cryptosporidia lacks both the Krebs cycle and the cytochrome-based respiration chain (e.g., *C*. *parvum* and *C*. *hominis*); whereas, others possess only the former but lack the latter (e.g., *C*. *muris*). Therefore, this genus of parasites relies mainly, if not solely, on glycolysis to produce ATP. To facilitate this “anaerobic metabolism”, *Cryptosporidium* possesses an L-lactate dehydrogenase (LDH) [EC 1.1.1.27], two alcohol dehydrogenases (ADHs), and an acetyl-CoA synthetase, which potentially produce lactic acid, alcohol, or acetic acid as organic end products [9]. Among these enzymes, LDH is known to be of the bacterial-type, likely derived from malate dehydrogenase (MDH) by a very recent gene duplication event [12]. LDH is considered to be a drug target in some parasites, including the apicomplexans *Plasmodium* and *Toxoplasma*, and in the anaerobic protozoa, *Giardia lamblia*, *Trypanosoma cruzi*, and *Entamoeba histolytica* [13, 14].

In the present study, we show that the *C*. *parvum* LDH (CpLDH) protein is distributed in the cytosol of free sporozoites and merozoites, but is then transferred to the PVM during intracellular development, indicating that in this parasite, the PVM is involved in lactate-fermentation. We also characterized the enzyme kinetic features of CpLDH and demonstrate that two known LDH inhibitors, gossypol and FX11, can inhibit both enzymatic activity and parasite growth *in vitro*.

## Results and Discussion

### CpLDH is a cytosolic protein in extracellular parasites, but is associated with the PVM during intracellular development

**Fig 1:**
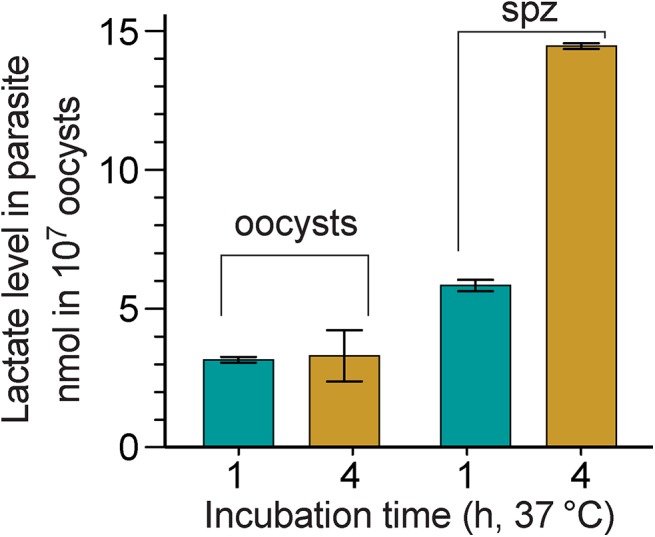
Lactate produced by *C*. *parvum* oocysts and free sporozoites. Oocysts were removed from refrigeration (4°C) and incubated at 37°C for 1 and 4 h, respectively. Sporozoites (spz) were prepared by excystation as described, and then incubated at 37°C for 1 and 4 h, respectively. Lactate levels released from 10^7^ oocysts or 4×10^7^ sporozoites are expressed in nanomolar amounts; means ± SD (n = 3) from one representative of three independent experiments.

We have previously used *C*. *parvum* microarray and qRT-PCR to show that the *CpLDH* gene is highly expressed in oocysts and free sporozoites, suggesting that pyruvate fermentation might be critical to these extracellular parasite stages [15]. To determine whether CpLDH is a metabolically active enzyme in the parasite, we measured the levels of lactate released by *C*. *parvum* oocysts and free sporozoites. We detected levels ranging from 3.1–14.4 nmol per 10^7^ oocysts or per 4×10^7^ sporozoites when these are incubated at 37°C for 1 to 4 h (Fig 1), confirming that lactate is released by *C*. *parvum* oocysts and sporozoites. A longer 4 h incubation increased the amount of lactate released by free sporozoites by 2.5-fold (i.e., from 5.8–14.4 nmol), but not by oocysts (i.e., from 3.15–3.29 nmol), suggesting that free sporozoites, after being excystated from oocysts, are more metabolically active than oocysts. Based on the size of sporozoites (~1×5 μm), we estimated that intracellular lactate concentrations in sporozoites could range from 19–91 mM if this metabolite is not released from, but rather, accumulates in the parasite (vs. ~1.3 mM in human normal bloods [16]).

**Fig 2:**
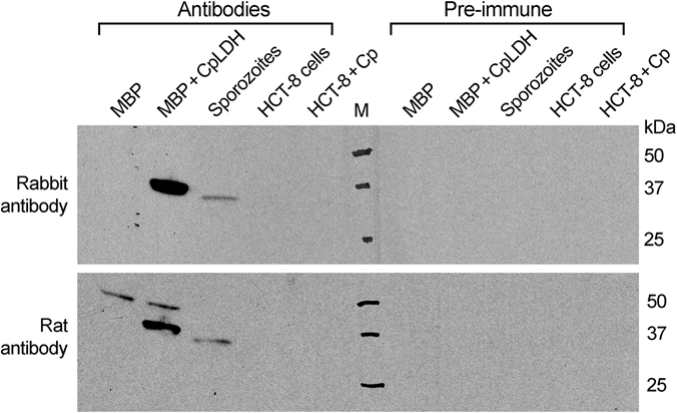
Western blot detection of CpLDH. The assays were performed using rabbit and rat polyclonal antibodies raised against a synthetic peptide or the recombinant CpLDH protein, respectively. Samples include recombinant maltose-binding protein (MBP), cleaved MBP-CpLDH fusion protein (MBP+CpLDH), and protein extracts from *C*. *parvum*-free sporozoites and HCT-8 cells infected with *C*. *parvum* for 18 h. Uninfected cells cultured in parallel were used as control. No immunoreactive bands are observed in samples probed with pre-immune sera. All antibodies, as well as pre-immune sera, were affinity purified as described in the Materials and Methods.

To investigate the distribution of the CpLDH protein in the parasite, we produced a rabbit polyclonal antibody against a CpLDH-specific peptide and a rat polyclonal antibody against the recombinant CpLDH protein. Antibodies were affinity-purified using the corresponding antigens (i.e., peptide and recombinant maltose-binding protein (MBP)-CpLDH fusion protein). Western blot analysis showed that neither antibody was cross-reactive with any host cell proteins, and both were able to recognize recombinant CpLDH protein. These antibodies detect bands at ~37 kDa and at ~34 kDa from whole proteins extracted from free sporozoites (Fig 2). These protein sizes agree with the theoretical masses of native CpLDH (33.9 kDa) and the recombinant CpLDH containing a few extra linker amino acids (37 kDa). MBP protein was labeled by the rat antibody, but not by the rabbit antibody, as the former was produced by immunizing rabbits with uncleaved MBP-CpLDH in first immunization, and subsequently with cleaved CpLDH in booster shots, while the later was raised against a peptide antigen. The presence of antibodies recognizing the bacterial MBP in the rat serum would not, however, complicate the subsequent immunofluorescence staining, due to the lack of MBP protein in parasite and human cells. Affinity-purified pre-immune sera produced no signals in all samples (Fig 2). These observations confirm the antibody specificity and the presence of the CpLDH protein in the parasite.

**Fig 3:**
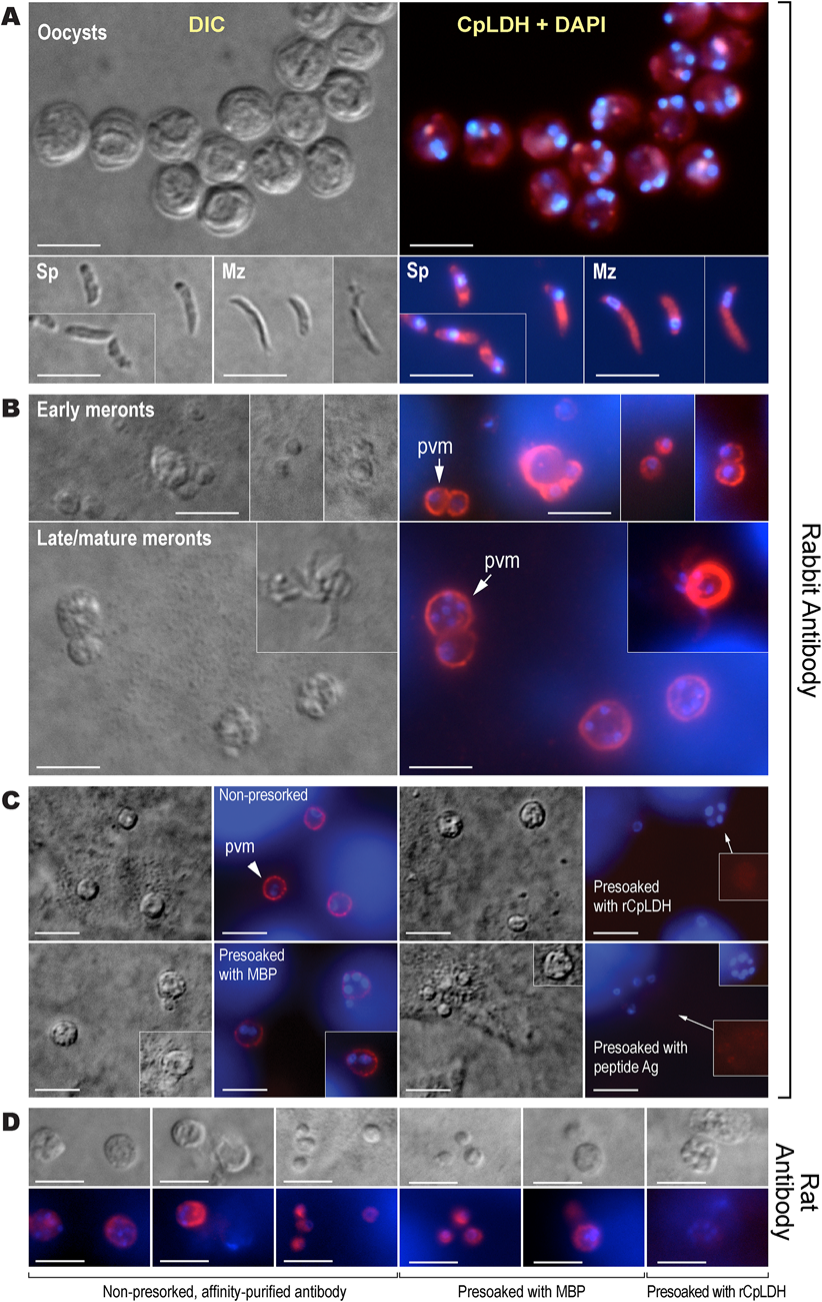
Immunofluorescence microscopic detection of CpLDH in different *C*. *parvum* life cycle stages. (A) Cytosolic distribution CpLDH in the extracellular parasite stages, including oocysts, sporozoites (Sp), and merozoites (Mz), using rabbit anti-CpLDH antibody. (B) Association of CpLDH with the parasitophorous vacuole membrane (PVM) during the parasite intracellular developmental stages, including early and mature meronts, using rabbit anti-CpLDH antibody. (C) Confirmation of the specificity of CpLDH detection in the PVM using primary antibodies that were untreated (non-presoaked) or presoaked with either the maltose-binding protein (MBP), recombinant CpLDH (rCpLDH), or synthetic peptide antigen (Ag). Fluorescence signals were eliminated by presoaking antibody with rCpLDH and synthetic antigen (right panel), but not with MBP (lower left panel). Insets on the right panel showed over-exposed images (red channel). (D) Detection of CpLDH protein in intracellular parasites using rat anti-CpLDH antibodies that were untreated or presoaked with either MBP or recombinant CpLDH (rCpLDH). All antibodies, including pre-immune sera, were affinity-purified as described in the Materials and Methods. No fluorescence signals were observed using rabbit and rat pre-immune sera (S1 Fig and S2 Fig). DIC, differential interference microscopy; CpLDH+DAPI, superimposed images of CpLDH labeled with TRITC and nuclei counterstained with DAPI. Bar = 5 μm.

**Fig 4:**
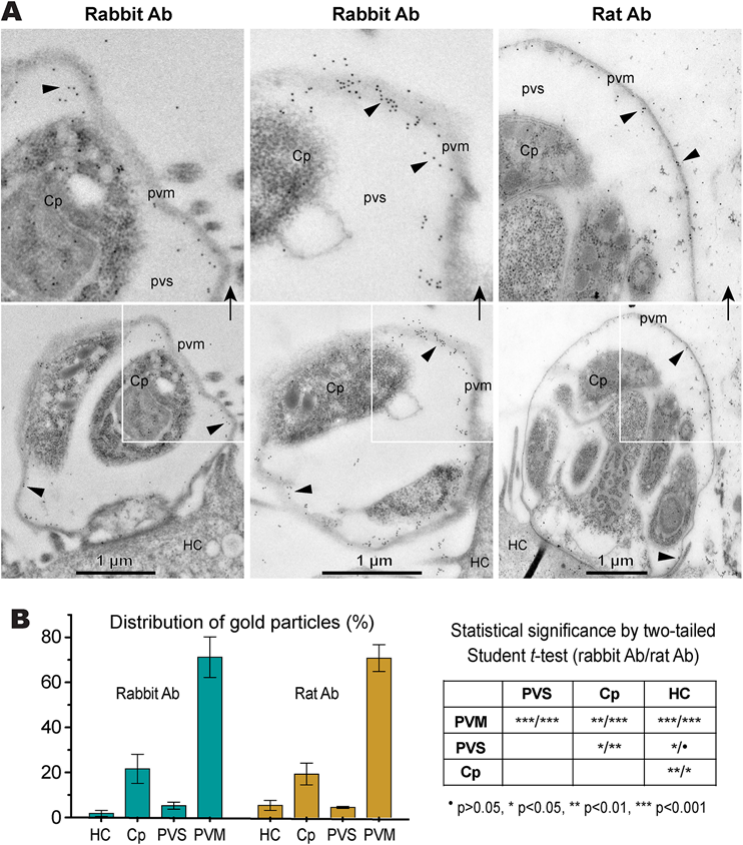
Subcellular distribution of CpLDH protein in intracellular *C*. *parvum* by immunoelectron microscopy (IEM). (A) Electron micrographs showing the distribution of CpLDH in intracellular parasites. Affinity-purified rabbit and rat antibodies (Ab) were used as primary antibodies. Colloidal gold beads were mainly distributed along the inner side of the parasitophorous vacuole membrane (PVM), although some gold beads were observable in intracellular parasites (Cp). Minimal numbers of particles were present in the host cell (HC) and parasitophorous vacuolar space (PVS). Arrowheads indicate example gold beads. Regions from the lower panel are enlarged in the upper panels, as indicated by arrows. (B) Bar chart showing the distribution of gold particles distributed in four types of cellular structures. Gold particles were manually counted, converted to the number of particles/μm^2^ and expressed as the percentage of total in each dataset. Means ± SD (n = 3) were calculated from three IEM images for each dataset. Statistical significance was assessed by two-tailed Student *t*-test.

Immunofluorescence microscopy using the rabbit antibody detected CpLDH in the cytosol of sporozoites within oocysts, in free sporozoites, and in merozoites (Fig 3A). However, during parasite intracellular development, CpLDH was found to be associated with the PVM (Fig 3B). While weak fluorescence signals were sometimes observed in intracellular parasites, these signals were insignificant in comparison with those from the PVM. No signals were observed in parasites or in host cells when using pre-immune sera (S1 Fig). We also performed immunofluorescence labeling using primary rabbit antibody that was presoaked with either synthetic peptide antigen, recombinant CpLDH, or MBP. We found that pretreatment with recombinant CpLDH protein or synthetic peptide antigen completely eliminated labeling of the PVM; whereas, presoaking with MBP had no effect on PVM labeling (Fig 3C). Similarly, the rat antibody also labeled proteins on PVM, and these signals could be eliminated by presoaking the antibodies with the CpLDH protein, but not with MBP (Fig 3D). The distribution of CpLDH in the PVM was further confirmed by immunoelectron microscopy, in which the majority of the colloidal-gold particles were observed along the inner side of the PVM (Fig 4A). In agreement with the immunofluorescence microscopy data, some gold particles were also present in intracellular parasites, but these were much fewer than those on, or near, the PVM. When gold particles were manually counted and expressed as number of particles/μm^2^, 71.2% (± 9.0%) and 70.8% (± 6.0%) of the total particles were present on or along the inner side of PVM when stained with rabbit and rat antibodies (vs. 21.7%–19.3% in the parasite merozoites, 5.3%–4.5% in the parasitophorous vacuolar space and 1.8%–5.4% in the host cells) (Fig 4B).

### The CpLDH-catalyzed reaction favors the formation of lactate and is inhibited by gossypol and FX11

**Fig 5:**
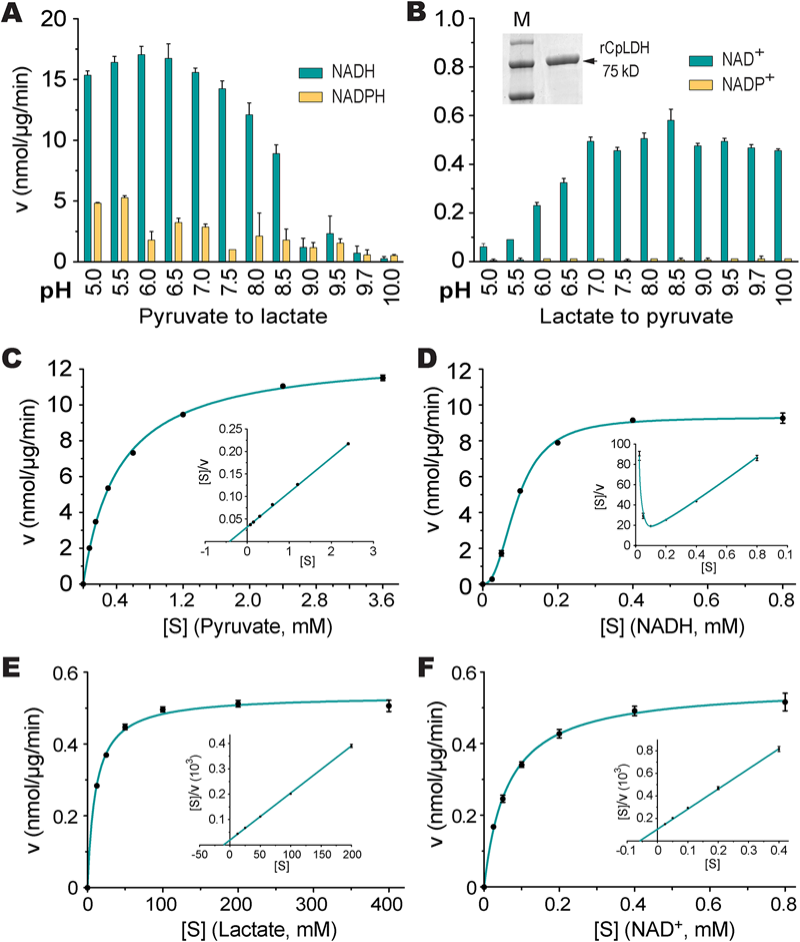
CpLDH enzyme kinetics. (A, B) Effects of pH value on the activity of CpLDH in the forward and reverse reactions. CpLDH prefers using NADH/NAD^+^ to convert pyruvate to lactate at conditions near neutral pH. Inset shows SDS-PAGE gel image of recombinant CpLDH (rCpLDH) used in these assays. (C—F) Activities of CpLDH on substrates, including pyruvate and lactate, and utilizing the cofactors NADH and NAD^+^. Insets show the Hanes-Woolf plots of the same datasets. Kinetic parameters are summarized in Table 1. S, substrate as indicated; v, velocity of the reaction. Means ± SD (n≥3) from one representative of at least three independent experiments.

To determine the biochemical properties of CpLDH, this enzyme was expressed as an MBP-fusion protein and purified to homogeneity (Fig 5B, inset). We observed that recombinant CpLDH was capable of catalyzing reactions in both directions (i.e., from pyruvate to lactate and vice versa). However, it favors the conversion of pyruvate to lactate, showing a >25-fold smaller *K*_m_ and a >24-fold larger *V*_max_ on pyruvate than on lactate (Table 1; Fig 5A–5F). This protein also displayed the highest activity near neutral pH values and preferred using NADH/NAD^+^ to NADPH/NADP^+^ (Fig 5A and 5B). CpLDH was found to act as an allosteric enzyme, with positive cooperativity on NADH (Hill coefficient = 2.4) (Fig 5D), but displayed typical Michaelis-Menten kinetics on pyruvate, lactate, and NAD^+^ (Fig 5C, 5E and 5F). The kinetic parameters we observed for this enzyme were comparable to those reported for the LDHs from *Plasmodium falciparum* and *Toxoplasma gondii* [17–19].

**Table 1:** Kinetic parameters of CpLDH on different substrates and with different cofactors.

Parameter	Pyruvate	NADH	Lactate	NAD^+^
*K*_m_ or *K*_0.5_ (μM)	427	92	10,830	62
*V*_max_ (nmol·μg^-1^·min^-1^)	12.89	9.33	0.53	0.56
*K*_cat_ (s^-1^)	16.1	11.7	0.66	0.70
*K*_cat_/*K*_m_ (s^-1^·M^-1^)	3.77 × 10^4^	1.27 × 10^5^	6.12 × 10^1^	1.13 × 10^4^
Hill coefficient		2.4		

**Fig 6:**
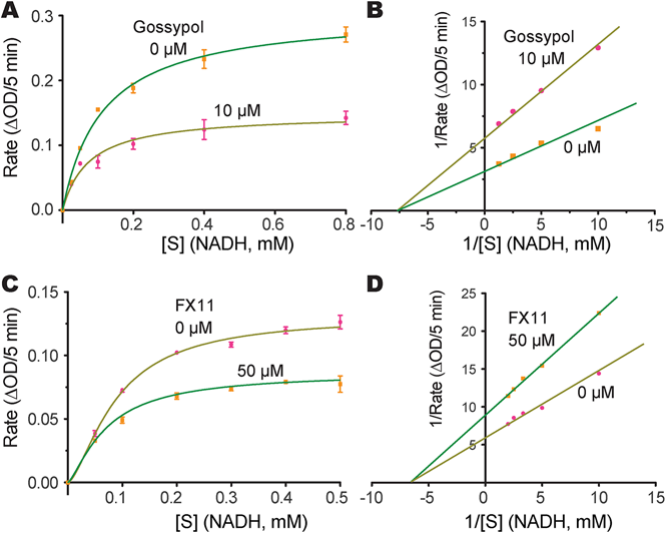
Inhibition constants of gossypol and FX11 against CpLDH. Michaelis-Menten curves (A, C) and corresponding Lineweaver-Burk plots (B, D) to determine the inhibition constants of gossypol (A, B) and FX11 (C, D) on CpLDH in respect to NADH. The reactions were conducted in 50 mM Tris-HCl buffer (pH 7.0), 2.4 mM pyruvate and varying concentrations of NADH. Separate experiments were performed using fixed amounts of pyruvate (2.4 mM) and NADH (250 μM) in the presence of various concentrations of gossypol and FX11 for determining the IC_50_ values (S3 Fig). Means ± SD (n ≥3) were calculated from one representative of at least three independent experiments.

We next evaluated the effects of two known LDH inhibitors, gossypol and FX11, on the enzymatic activity of CpLDH. Gossypol is a polyphenolic compound derived from cotton plants that acts as an inhibitor of LDHs, including those from humans, *Plasmodium*, and *Toxoplasma* [20, 21], while FX11 was recently discovered to be a selective inhibitor of human LDH-A, displaying *K*_i_s on HsLHD-A and HsLDH-B of ~8 μM and >90 μM, respectively [22]. We found that both compounds acted as noncompetitive inhibitors on CpLDH, with respect to NADH (Fig 6). For gossypol, the *K*_i_ and IC_50_ values were 14.8 μM and 21.0 μM, respectively (Table 2). This *K*_i_ value was slightly larger than, but comparable to, those reported for PfLDH (0.7 μM), TgLDHs (1.1–6.1 μM), and human LDHs (1.4–4.2 μM) [17, 18, 20], suggesting that, similar to PfLDH and TgLDHs, CpLDH is sensitive to inhibition by gossypol, and possibly to derivatives of this compound as well. FX11 was also able to inhibit CpLDH enzymatic activity at low micromolar levels, but it was less effective on CpLDH than on HsLHD-A (i.e., the *K*_i_ and IC_50_ values on CpLDH were 55.6 μM and 65.0 μM, respectively) (Table 2).

**Table 2:** Inhibition constants and anti-cryptosporidial activities of gossypol and FX11.

Parameter	Gossypol	FX11
Inhibition on CpLDH and HsLDH enzymes (μM)		
CpLDH (*K*_i_)	14.8	55.6
CpLDH (IC_50_)	21.0	65.0
HsLDH-A (*K*_i_)*	1.9	8
HsLDH-B (*K*_i_)*	1.4	>90
Inhibition on the growth of parasite or host cell *in vitro* (μM)		
Growth of *C*. *parvum* (qRT-PCR)	11.8	39.5
Growth of HCT-8 cells (MTT)	51	87

* These values were acquired from Yu *et al*., 2001 and Le *et al*., 2010 [20, 22].

### LDH inhibitors interfere with *C*. *parvum* growth *in vitro*

**Fig 7:**
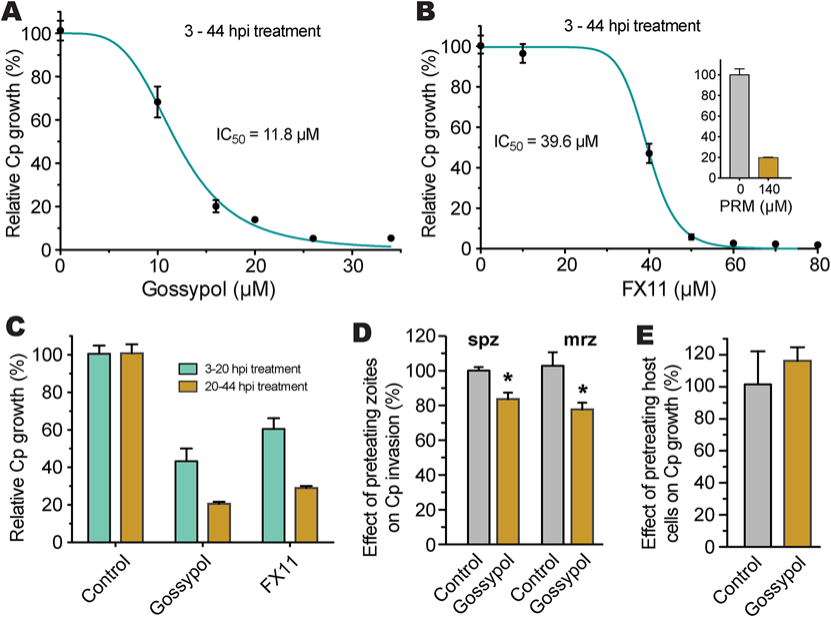
Efficacies of gossypol and FX11 on the growth of *C*. *parvum* in HCT-8 cells. (A, B) Inhibition of *C*. *parvum* growth *in vitro* by gossypol and FX11 in a standard 44-h infection assay, in which intracellular parasites were treated for 3–44 h post-infection (hpi). Paromomycin at 140 μM was used as positive control. (C) Effects of gossypol (16 μM) and FX11 (60 μM) on the first and second asexual developmental cycles (merogony). Cultured parasites were treated with specified inhibitors for 3–20 h post-infection (hpi) and for 20–44 hpi, respectively, and parasite growth was evaluated at the end of each treatment. (D) Effects of pretreatment of free sporozoites (spz) and type I merozoites (mrz) with gossypol on their attachment and invasions into host cells (40 min pretreatment + 3 hpi assay). (E) Effect of pretreatment of host cells by gossypol on parasite infection, in which HCT-8 cells were pretreated with gossypol (16 μM, 24 h) followed by 3 h parasite infection in the absence of inhibitor. Anti-cryptosporidial activities were determined by a qRT-PCR-based assay. Means ± SD (n ≥3) from one representative of at least three independent experiments. An asterisk indicates statistically significant difference between the sample and corresponding control by two-tailed Student *t*-test (*p*<0.05).

We further tested the effect of gossypol and FX11 on the *in vitro* growth of *C*. *parvum* in HCT-8 cells using a qRT-PCR assay that we previously developed [15, 23]. We performed 44 h infection assays, in which oocysts were incubated for 3 h in a drug-free medium to allow excystation and invasion before inhibitors were added into the culture via a medium exchange. Subsequently, we found that both inhibitors exhibit anti-cryptosporidial activity at low micromolar concentrations (i.e., IC_50_ for gossypol and FX11 were 11.8 μM and 39.6 μM, respectively) (Fig 7A and 7B). The positive control, paromomycin at 140 μM, inhibited parasite growth by 80.3% as expected (Fig 7B, inset). Gossypol and FX11 both displayed low to moderate cytotoxicity on cancerous HCT-8 host cells at all tested concentrations; their IC_50_ values on HCT-8 cells, as determined by MTT assay, were 51 μM and 87 μM, respectively (Table 2). The anti-cryptosporidial IC_50_ value for gossypol was comparable to those reported for the same compound against *P*. *falciparum* (7–13 μM) and *T*. *gondii* (between 5–10 μM) [18, 24]. Conversely, to the best of our knowledge, this is the first report of anti-parasitic activity for FX11. The anti-cryptosporidial efficacy of FX11 was lower than that of gossypol, which was well-correlated with their *K*_i_ values on CpLDH (Table 2).

To evaluate the effects of inhibitors on individual asexual developmental stages, gossypol (16 μM) and FX11 (60 μM) were applied to intracellular parasites between 3 to 20 h post-infection (hpi) (corresponding to the first generation of merogony), and between 20 to 44 hpi (corresponding to the second generation of merogony and some gametogenesis). In this assay, both asexual developmental stages could be inhibited, but the efficacies were higher against the second generation of merogony development (Fig 7C). We observed that pretreatment of excystated sporozoites and type I merozoites with 16 μM gossypol for 40 min at room temperature reduced parasite invasion (3-hpi assay) by 17% and 25%, respectively (Fig 7D). The reductions were statistically significant (*p*<0.05 by Student’s *t*-test), but less dramatic than expected considering that CpLDH is expressed at the highest levels in these motile stages. This might be explained by the fact that: 1) gravity could help sporozoites and merozoites settle down to the bottom of the culture wells to reach the host cells, so parasites might only need minimal energy to reach to the host cells, 2) the invasion process was rapid, and/or 3) the CpLDH levels were too high to be significantly inhibited by gossypol.

It is possible that gossypol and FX11 could also inhibit host cell LDH and other metabolic pathways, which might in turn affect parasite infection and growth. To address this possibility, HCT-8 cells were pretreated with gossypol (16 μM) for 24 h and then infected with *C*. *parvum* sporozoites in the absence of inhibitor. We observed that pretreatment of host cells with 16 μM gossypol had no inhibitory effect on parasite infection (Fig 7E). Rather, a slight, albeit non-statistically significant, increase in parasite infection was observed.

**Fig 8:**
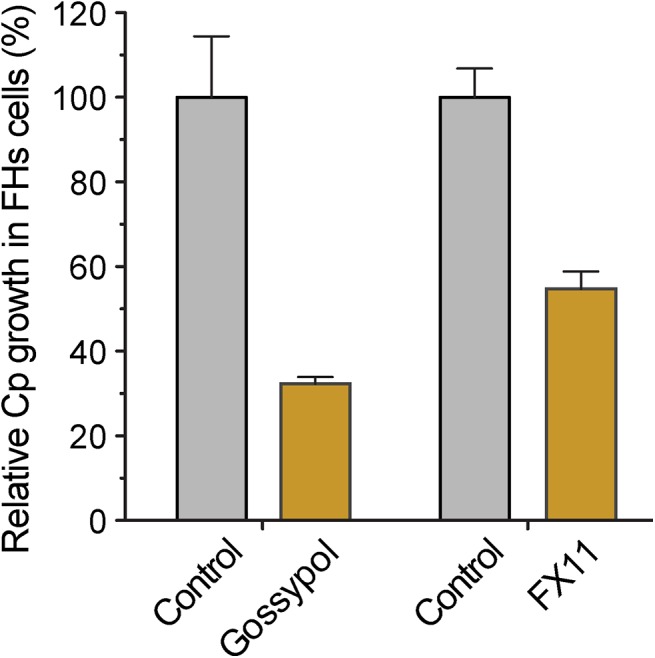
Effects of LDH inhibitors on the growth of *C*. *parvum* in primary enterocytes. Parasites were cultured in FHs 74 Int primary enterocytes for 44 h, and anti-cryptosporidial activities of gossypol (10 μM) and FX11 (60 μM) were determined by a qRT-PCR-based assay.

Because the glycolytic pathway of cancer cells might be more susceptible to modulation by LDH inhibitors, we performed a 44-h infection assay using FHs 74 Int primary fetal enterocytes (ATCC #CCL-241). Here, we observed that gossypol (10 μM) and FX11 (60 μM) inhibited the growth of *C*. *parvum* by 67.7% and 45.3%, respectively (Fig 8). This primary cell line is a highly valuable alternative to the cancer cells for studying *Cryptosporidium* infection *in vitro*. However, the conditions for growing *C*. *parvum* in FHs 74 Int cells require further optimization, as parasite growth was much less efficient in these cells than in the commonly used HCT-8 and Caco-2 cells.

## Discussion

In the present study, we found that the CpLDH protein is cytosolic during the motile, extracellular, stages of parasite growth, but associates with the PVM during the intracellular development. The PVM is a unique membrane structure, which hosts the intracellular development of apicomplexan parasites and facilitates interactions between these parasites and host cells. The *Cryptosporidium* PVM is unique from those of other apicomplexans, such as *Plasmodium*, *Toxoplasma*, and *Eimeria*, in that it localizes on the top of host cells, rather than in the cytosol. Therefore, *Cryptosporidium* is an intracellular, but extracytoplasmic, parasite. The protein composition of the cryptosporidial PVM is poorly understood. Currently, only a few proteins involved in fatty acid metabolism have been localized to the PVM, such as the long-type fatty acyl-CoA binding protein (CpACBP) [25], an oxysterol-binding protein-related proteins (CpORP1) [25], and the long chain fatty acid elongase (CpLCE) [26]. The discovery that CpLDH is a PVM-association protein suggests that this unique structure participates not only in fatty acid metabolism, but also in lactate fermentation. Since lactate cannot be reutilized by *Cryptosporidium*, we speculate that the PVM localization of CpLDH facilitates the quick release of lactate into host cells and/or the environment to eliminate the potential detrimental effect caused by the accumulation of this compound in the parasite.

**Fig 9:**
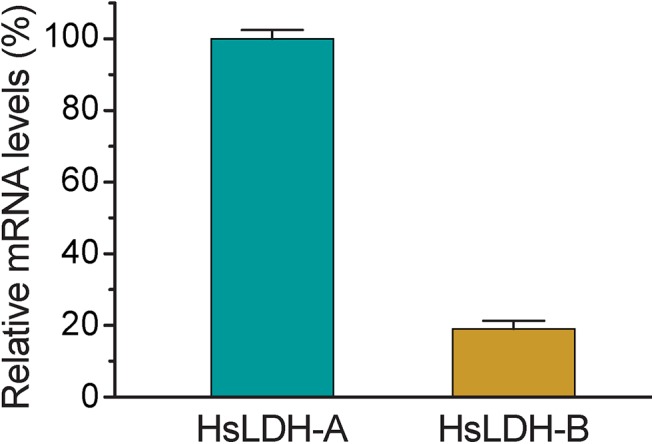
Relative mRNA levels of two human LDH isoforms in host cells. Total RNA was isolated from HCT-8 cells to determine the levels of mRNA of HsLDH-A and HsLDH-B and those of 18S rRNA by qRT-PCR. The levels of HsLDH isoforms were expressed in relative to the host cell 18S rRNA.

We also characterized the biochemical activity of CpLDH and observed that the LDH inhibitors gossypol and FX11 also inhibit both CpLDH enzymatic activity and the growth of *C*. *parvum in vitro* at low micromolar concentrations. Gossypol and FX11 are mammalian LDH inhibitors and have been explored as potential therapeutics against cancer cells, which rely more heavily on aerobic glycolysis to survive [27]. Of the two, gossypol may act on other targets in host cells, including steroid dehydrogenase, telomerase, calcineurin phosphatase, aromatase, ribonucleotide reductase arachidonate lipoxygenases, adenylate cyclase, catechnol-*O*-methyltransferase, phospholipase A2, and apurinic/apyrimidinic endonuclease 1/redox enhancing factor-1 (APE1) [28–35]. FX11 was recently discovered as a selective LDH-A inhibitor [22, 36]. Because many cancer cells mainly rely on LDH-A, inhibition by FX11 is considered to be an achievable, and tolerable, treatment for LDH-A-dependent tumors. In the case of HCT-8 cells, which are commonly used for studying *Cryptosporidium* infection *in vitro*, the mRNA level of LDH-A is >5-fold higher than that of LDH-B (Fig 9). Therefore, it is possible that gossypol and FX11 might inhibit the growth of *C*. *parvum in vitro* not only *via* direct action on the parasite LDH, but also by altering the host cell metabolism. However, this seems unlikely as pretreatment of host cells with gossypol at 16 μM for 24 h had no effect on the growth of *C*. *parvum* (Fig 7E), suggesting that the physiological changes induced by gossypol only minimally contribute to its anti-*Cryptosporidium* activity. Additionally, we also observed anti-*Cryptosporidium* activity for both gossypol and FX11 when using FHs 74 Int primary cells, which are less sensitive than cancer cells to the inhibition of LDH activity (Fig 8). Collectively, these observations indicate that the anti-*Cryptosporidium* activity of gossypol and FX11 is largely due to their inhibitory activity against CpLDH.

It is understood that the ultimate validation of CpLDH as a drug target can only be achieved using genetic tools for knockout or knockdown of genes of interest in *Cryptosporidium*. More recently, the first successful genetic modification of *C*. *parvum* was reported using CRISPR/Cas9 [37]. Although the current transfection system requires to propagate parasites in mice and remains to be further developed into a routine technique, it raises a hope that CpLDH and other potential drug targets in *C*. *parvum* may be genetically validated in the very near future. It is also noticeable that the observed anti-*Cryptosporidium* activities of gossypol and FX11 *in vitro* are not strong enough for drug development of these specific compounds. Nonetheless, the present data, together with the fact that *C*. *parvum* relies on glycolysis for producing ATP due to its lack of the Krebs cycle and cytochrome-based respiratory chain, support the notion that the bacterial-type CpLDH is worth exploring as a potential target for the development of anti-cryptosporidial therapeutics.

## Materials and Methods

### Parasites

The IOWA-1 strain of *C*. *parvum* was used in all experiments. Parasite oocysts were purchased from Bunch Grass Farm (Deary, Idaho, USA) and stored in phosphate-buffered saline (PBS) at 4°C until use. Oocysts were treated with 10% Clorox in ice for 8 min, washed 5–8 times with water by centrifugation, and purified by a Percoll gradient centrifugation protocol [38]. For all parasite experiments, we used oocysts that were less than 3 months old (since harvest). Free sporozoites were prepared by an excystation procedure, in which oocysts were incubated in PBS (pH 7.5) containing 0.25% trypsin and 0.5% taurodeoxycholic acid at 37°C for 1 h, followed by three or more washes with PBS. Free merozoites (type I) were collected from the culture medium after *in vitro* culture of parasites for ~20 h in HCT-8 cells (also see more detailed *in vitro* cultivation procedures below).

### Antibody preparation and western blot analysis

Rabbit anti-CpLDH antibody was prepared commercially in two specific pathogen-free rabbits, using a CpLDH-specific synthetic peptide, ^297^KLLGESINEVNTIS^310^, conjugated to keyhole limpet hemocyanin at the N-terminus as antigen, by a standard immunization protocol (GenScript [Piscataway, NJ]). A second anti-CpLDH antibody was produced in five specific pathogen-free rats using recombinant CpLDH protein as antigen by Alpha Diagnostic International (San Antonio, TX), in which the first immunization were performed with the MBP-CpLDH fusion protein, and booster shots were administered with cleaved and purified CpLDH. Polyclonal antibodies were affinity purified using synthetic peptide conjugated to the agarose resin (rabbit antibody) or the recombinant CpLDH protein conjugated onto nitrocellulose membrane (rat antibody) as described [39]. Pre-immune sera were similarly affinity-purified in parallel using corresponding antigens (i.e., peptide and recombinant protein for rabbit and rat antibodies, respectively) from the same volumes of sera, and eluted into the same amount of elution buffer as used for antisera.

For western blot analysis, *C*. *parvum* oocysts and sporozoites were prepared in RIPA buffer containing protease inhibitor cocktail (Sigma-Aldrich, St. Louis, MO). Oocysts were disrupted using five freeze-thaw cycles, while free sporozoites prepared by the excystation procedure were directly lysed in RIPA buffer. Parasite lysates (2.0×10^7^ oocysts or 7.0×10^7^ sporozoites per lane) were fractionated by 10% SDS-PAGE and transferred onto nitrocellulose membranes. The blots were treated in 5% BSA in TBST buffer (containing 50 mM Tris-HCl (pH 7.5), 150 mM NaCl and 0.05% Tween 20), incubated with the purified anti-CpLDH polyclonal antibody (1:500 dilution in 5% BSA-TBST), and then treated with horseradish peroxidase-conjugated goat-anti-rabbit or anti-rat IgG antibody (1:5000 dilution). The blots were then visualized using an enhanced chemiluminescence reagent (Sigma-Aldrich).

### Indirect immunofluorescence microscopy and immunoelectron microscopy

*C*. *parv*um oocysts, sporozoites, and type I merozoites were fixed in 4% formaldehyde for 20 min, washed with PBS, and then applied onto poly-L-lysine coated coverslips. After these were air-dried at ambient temperature, cells were permeabilized with cold methanol:acetone (V:V = 1:1) for 5 min at -20°C. For the intracellular parasite stages, host cells infected with *C*. *parvum* were fixed in 4% formaldehyde for 30 min. After three washes in PBS, excess formaldehyde was quenched with 50 mM NH_4_Cl for 15 min. Fixed cells on coverslips were washed and permeabilized with 0.1% Triton X-100 and 0.05% SDS in PBS for 5 min. Samples were then incubated with affinity-purified anti-CpLDH antibody (1:50 dilution in 1% FBS-PBS) for 1 h, followed by 3 washes with 1% FBS-PBS (5 min each) and incubation with TRITC-conjugated goat anti-rabbit IgG antibody (1:400 dilution) in PBS for 1 h. After three more washes with PBS, samples were mounted on glass slides in a SlowFade mounting medium, containing 4',6-diamidino-2-phenylindole (DAPI) for counter-staining of nuclei (Molecular Probes/Invitrogen). All incubations and washes were performed at room temperature or as specified. Cells labeled with fluorescent molecules were examined with an Olympus BX51 research microscope with appropriate filter sets. Images were captured with a Retiga SRV CCD Digital Camera (QImaging) and uniformly manipulated with Adobe Photoshop CS6 for signal contrast and intensity.

For immunoelectron microscopy, intracellular parasites were similarly prepared, but grown in LabTek Permanox chamber slides for 18 hpi. Infected monolayers were washed in PBS and fixed in 4% paraformaldehyde, mixed with 0.1% glutaraldehyde in PBS for 15 h at 4°C. Fixed samples were washed with 0.05 M maleate buffer, containing 0.5 mM CaCl_2_ and 2% sucrose, incubated for 30 min at 20°C in 0.5% uranyl acetate in maleate buffer, and then washed again in maleate buffer. Samples were dehydrated in a gradient ethanol series, then infiltrated with LR White acrylic resin and cured at 50°C for 13 h. Selected segments of embedded samples were transferred to LR White in gelatin capsules and cured at 50°C for an additional 14 h. Thin sections were produced using a diamond knife on a Leica EM UC6 ultramicrotome and mounted on film-coated grids. Sections on grids were blocked sequentially in blocking buffer I (100 mM glycine in TBS) and II (2.5% BSA/0.2% cold water fish gelatin), and then incubated with primary antibody at 4°C overnight. After washes, samples were labeled with goat anti-rabbit IgG secondary antibodies conjugated with 10 nm gold beads for 2 h at room temperature, washed again, and then post-stained briefly with 2% uranyl acetate and Reynold’s lead citrate. Colloidal gold-labeled thin sections were examined with a Philips Morgagni 268 transmission electron microscope (FEI Company, Hillsboro, OR) at an accelerating voltage of 80 kV. Digital images were recorded with a MegaViewIII digital camera operated with iTEM software (Olympus Soft Imaging Systems). The distribution of gold particles was manually counted, in which IEM images containing full-sized PVM structures were cropped into separate images representing four types of cellular structures (i.e., PVM, parasitophorous vacuolar space, parasite and host cell) using Photoshop CS6 Extended (Adobe Systems Inc., San Jose, CA). The area of each structure was measured for calculating the numbers of gold particles per square micrometer. The relative density of gold particles were expressed as the percentage of the total in each dataset. Three IEM images were measured for each type of antibody. Statistical significance was evaluated by two-tailed Student *t*-test for all individual pairs of structures.

### Detection of lactate released by *C*. *parvum* oocysts and sporozoites

The lactate released by parasite oocysts and sporozoites was detected using a lactate assay kit (Eton Bioscience Inc., San Diego, CA). In this assay, LDH converts lactate and NAD^+^ into pyruvate and NADH, and then a NADH-coupled enzyme reaction reduces a tetrazolium salt INT (2-(4-iodophenyl)-3-(4-nitrophenyl)-5-phenyl-2H-tetrazolium chloride) into formazan that exhibits an absorbance maximum at 490 nm. Oocysts (3×10^7^) removed from refrigeration (4°C) were washed twice with PBS and resuspended in 200 μL PBS. Free sporozoites were prepared from 3×10^7^ oocysts by an excystation procedure, as described above, and resuspended in 200 μL PBS. Both oocyst and sporozoite samples were subjected to centrifugation after incubation at 37°C for 1 and 4 h, respectively, from which 20 μL of supernatants were used for detecting lactate contents. The assay was carried out in 70 μL of reaction buffer in 384-well microplates. The reactions were initialized by adding 50 μL of lactate reaction solution into 20 μL sample solutions or lactate standards (20 to 640 μM). After incubation at 37°C for 1 h, the reactions were stopped by adding 50 μL of 0.5 M acetic acid. OD_490_ values were measured using a Multiskan spectrum spectrophotometer (Thermo Scientific, West Palm Beach, FL).

### Biochemical assays

The pMALc2x-CpLDH vector, described in a previous study, was used to express recombinant CpLDH protein in a Rosetta 2 strain of *Escherichia coli* [12]. Briefly, a single colony of transformed bacteria was inoculated into 50 mL LB media, containing ampicillin (50 μg/ml), chloramphenicol (25 μg/ml), and glucose (2 mg/ml), and this was allowed to grow overnight at 37°C. The overnight cultures were diluted with fresh medium (1:10 ratio) and incubated for ~2 h at 37°C until their OD_600_ reached ~0.5. Isopropyl-1-thio-β-D galactopyranoside (IPTG) was then added to the cultures at a final concentration of 0.3 mM to induce protein expression and bacteria were incubated for ~16 h at 16°C for protein overexpression. Bacterial pellets were collected by centrifugation, and MBP-fusion protein was purified by amylose resin-based affinity chromatography, according to manufacturer’s protocols (New England Biolabs, Ipswich, MA). The MBP-tag alone was similarly expressed, purified under the same conditions, and used as negative control.

All chemicals used in biochemical assays were purchased from Sigma-Aldrich or as specified. The activity of CpLDH against pyruvate and lactate was determined by monitoring the reduction of NADH at OD_340_ in a Multiskan Spectrum spectrophotometer (Thermo Scientific). Forward direction assays were performed in 200 μL reaction buffer, containing 50 mM Tris-HCl buffer (pH 8.0), 100 ng MBP-CpLDH1, 0.25 mM NADH, and 1.2 mM pyruvate. Reverse direction assays were similarly performed in 50 mM Tris-HCl buffer (pH 9.2), containing 500 ng MBP-CpLDH1, 1 mM NAD^+^, and 100 mM L-lactate. For testing the effect of pH on CpLDH activity, in the forward and reverse reactions, three buffer systems, Citric Acid–Na2HPO4 buffer (pH 5.0–6.5), Tris-HCl buffer (pH 7.0–9.0), and Sodium Carbonate–Sodium Bicarbonate buffer (pH 9.0–10.0), were used. Varied substrate and cofactor concentrations were used for determining the kinetic parameters (i.e., pyruvate at 20–3,600 μM, NADH at 25–800 μM, lactate at 12.5–400 mM, and NAD^+^ at 25–800 μM).

The effect of gossypol or FX11 on the ability of CpLDH to catalyze the conversion of pyruvate to lactate was examined in reactions containing 50 mM Tris-HCl buffer (pH 7.0), 2.4 mM pyruvate, varied concentrations of NADH, and 10 μM of either gossypol or 50 μM of FX11. In all experiments, MBP-tag only was used as a control for background subtraction. All assays were carried out in triplicate, and at least two independent assays were performed. GraphPad Prism (v5.0f) (http://www.graphpad.com) was used to calculate kinetic parameters with appropriate nonlinear regression models, including Michaelis-Mention and allosteric sigmoidal models for substrates/cofactors. Only reaction data in the linear range were used in computations, which typically occurred within a reaction time of 5 min. The *K*_i_ values for gossypol and FX11 were calculated by Lineweaver-Burke plot and by the Cheng-Prusoff equation for noncompetitive inhibitors [40].

### *In vitro* drug testing

HCT-8 cells (ATCC# CCL-244) were maintained as previously described [23]. Prior to each infection experiment, HCT-8 cells were seeded into 24-well flat bottom plates and allowed to grow overnight until they reached ~80% confluence. In a typical 44-h infection assay, *C*. *parvum* oocysts were added to wells at a parasite:host cell ratio of 1:2 (10^5^ oocysts/well). Cells receiving no infection, or sham infection with oocysts heat killed by pretreatment at 65°C for 30 min, were included as negative controls. After incubation for 3 h at 37°C to allow parasite excystation and invasion, uninfecting parasites were removed, and fresh medium containing 3.125 to 34 μM gossypol, or 10 to 80 μM FX11 was added. Medium containing 140 μM paromomycin was used as a positive control. Intracellular parasites were allowed to grow for additional 41 h (total 44 h growth time) before total RNA was isolated. The effect of inhibitors on different asexual developmental stages was similarly assayed with one dose of gossypol (16 μM) or FX11 (60 μM), in which infected cells were treated with inhibitors between 3–20 h and 20–44 h hpi, respectively. Total RNA was isolated at the end of each treatment. The cytotoxicity of both gossypol and FX11 against HCT-8 cells was evaluated by an MTT-based *in vitro* Toxicology Assay Kit (Sigma-Aldrich), as described elsewhere [41].

The effect of inhibitors on the attachment/invasion of free sporozoites and type I merozoites (*C*. *parvum* motile stages) was also evaluated. Sporozoites were prepared by an excystation procedure, while merozoites were collected from the culture supernatants as described above. Free sporozoites and merozoites were pretreated with 16 μM gossypol in culture medium at room temperature for 40 min, and then added to plates containing HCT-8 cells (10^4^ sporozoites and 10^3^ merozoites per well, respectively). After 3 hpi, uninfected parasites were removed by three washes with PBS, and the infected host cell monolayers were lysed for RNA isolation.

We also evaluated the effects of gossypol and FX11 on the growth of *C*. *parvum in vitro* in FHs 74 Int primary enterocytes (ATCC# CCL241). In this assay, host cells were similarly cultured as described above and inoculated with *C*. *parvum* oocysts at a parasite:host cell ratio of 1:2. After 3 h of invasion, free parasites in the medium were removed, and gossypol (10 μM) and FX11 (60 μM) were separately added into the culture with a medium exchange. Infected cells were allowed to grow for additional 41 h, followed by the isolation of total RNA. In all experiments, samples receiving no gossypol (untreated groups) were used as negative control. At least three biological replicates, plus two technical replicates were performed for each experiment.

Parasite loads were evaluated by qRT-PCR-based detection of the relative levels of parasite 18S rRNA as previously described [15, 23]. Briefly, total RNA was isolated from HCT-8 cells that were either uninfected or infected with parasites for various times, using the RNeasy Mini Kit (QIAGEN Inc., Valencia, CA). RT-PCR reactions were performed using a QIAGEN one-step RT-PCR QuantiTect SYBR green RT-PCR kit, using the following pairs of primers: Cp18S-1011F (5’ TTG TTC CTT ACT CCT TCA GCA C 3’) and Cp18S-1185R (5’ TCC TTC CTA TGT CTG GAC CTG 3’) for *C*. *parvum* 18S rRNA (Cp18S), and Hs18S-1F (5’ GGC GCC CCC TCG ATG CTC TTA 3’) and Hs18S-1R (5’ CCC CCG GCC GTC CCT CTT A 3’) for host cell 18S rRNA (Hs18S). Each reaction mixture (25 μl) contained 2 ng total RNA, 500 nM each primer, 10 nM fluorescein isothiocyanate (FITC), 0.25 μL RT master mix, and 1x QuantiTect SYBR green. Mixtures were incubated at 50°C for 30 min for synthesizing cDNA, heated at 95°C for 15 min to inactivate the reverse transcriptase, and then subjected to 40 thermal cycles of PCR amplification (95°C for 20 s, 58°C for 30 s, and 72°C for 30 s) in a CFX Connect real-time PCR detection system (Bio-Rad Laboratories, Hercules, CA). At least two replicate qRT-PCRs were performed for each sample, and all qRT-PCR reagents were loaded manually. Relative levels of parasite 18S rRNA were calculated by a ΔΔCT method with a general formula of 2^-ΔΔCT^, in which changes in threshold cycle (ΔCT) values between Cp18S and Hs18S were first determined by the equation C_T[Cp18S]_ − C_T[hs18S]_, followed by the calculation of ΔΔC_T_ between treated and untreated samples. Statistical significance on the relative levels of parasite 18S rRNA was evaluated by Student’s *t-*test.

### Relative expression levels of LDH isoforms in host cells

HCT-8 cells were seeded and cultured for overnight as described above for the isolation of total RNA as described above. The relative levels of the HsLDH-A and Hs-LDH-B genes were determined by qRT-PCR using the following primers: hLDHA-F376 (5’ GGG GCA CGT CAG CAA GAG GG 3’) and hLDHA-R488 (5’ AGC AAC TTG CAG TTC GGG CTG T 3’) (LDH-A) and hLDHB-F981 (5’ TGC CCG GGG ATT AAC CAG CGT 3’) and hLDHB-R990 (5’ GTC CTT CTG GAT GTC CCA CAG GGT 3’) (LDH-B). Levels of human 18S rRNA were used for normalization.

## Supporting Information

S1 Fig Immunofluorescence microscopy using rabbit pre-immune serum. Pre-immune serum was affinilty-purified in parallel with anti-serum (as described in the Materials and Methods), and used to label sporozoites, merozoites and meronts of *Cryptosporidium parvum*. No cross-reactivity was observed between the pre-immune serum and parasites or HCT-8 host cells. Only slightly higher than the background signals were observed when TRITC signals were over-exposed, but the patterns differed from those labeled with affinity-purified rabbit anti-serum. Bars = 5 μm.
(TIF)

S2 Fig Immunofluorescence microscopy using rat pre-immune serum. Pre-immune serum was affinilty-purified in parallel with anti-serum (as described in the Materials and Methods), and used to label early and late stages of *Cryptosporidium parvum* meronts. No cross-reactivity was observed between the pre-immune serum and parasites or HCT-8 host cells. Only slightly higher than the background signals were observed when TRITC signals were over-exposed, but the patterns differed from those labeled with affinity-purified rabbit anti-serum. Bars = 5 μm.
(TIF)

S3 Fig Inhibition curves of gossypol and FX11 on the CpLDH enzyme activities. Assays were performed using fixed amounts of pyruvate (2.4 mM) and NADH (250 μM) in the presence of various concentrations of gossypol and FX11.
(TIF)
