# Supplementary figures and images for: Cryptosporidium Lactate Dehydrogenase Is Associated with the Parasitophorous Vacuole Membrane and Is a Potential Target for Developing Therapeutics
Source: PLoS Pathog. 2015 Nov 12;11(11):e1005250. doi: 10.1371/journal.ppat.1005250 (PMC4642935; doi:10.1371/journal.ppat.1005250)

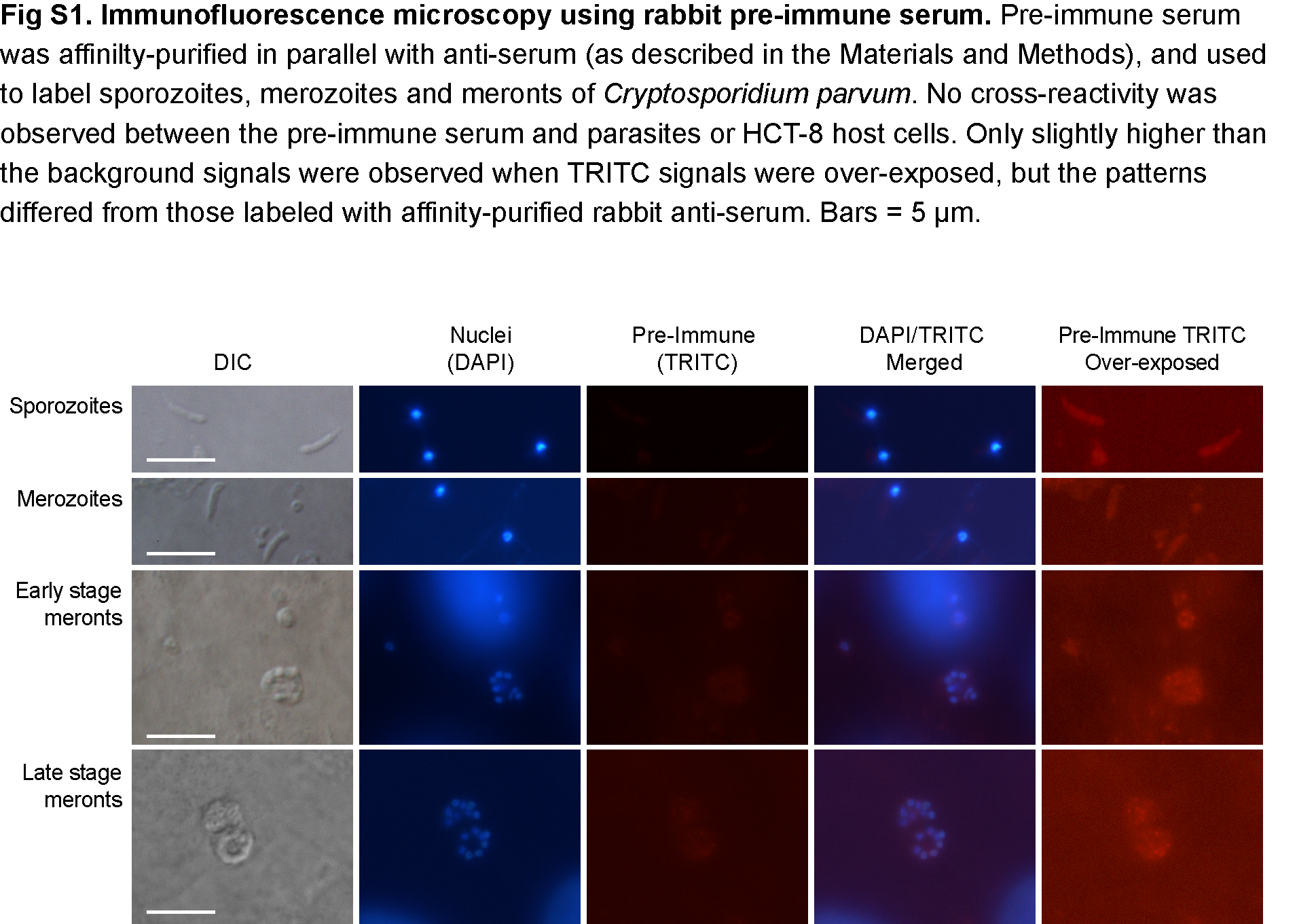

Supplement: S1 Fig — Pre-immune serum was affinilty-purified in parallel with anti-serum (as described in the Materials and Methods), and used to label sporozoites, merozoites and meronts of Cryptosporidium parvum. No cross-reactivity was observed between the pre-immune serum and parasites or HCT-8 host cells. Only slightly higher than the background signals were observed when TRITC signals were over-exposed, but the patterns differed from those labeled with affinity-purified rabbit anti-serum. Bars = 5 μm. (TIF) [file ppat.1005250.s001.tif]

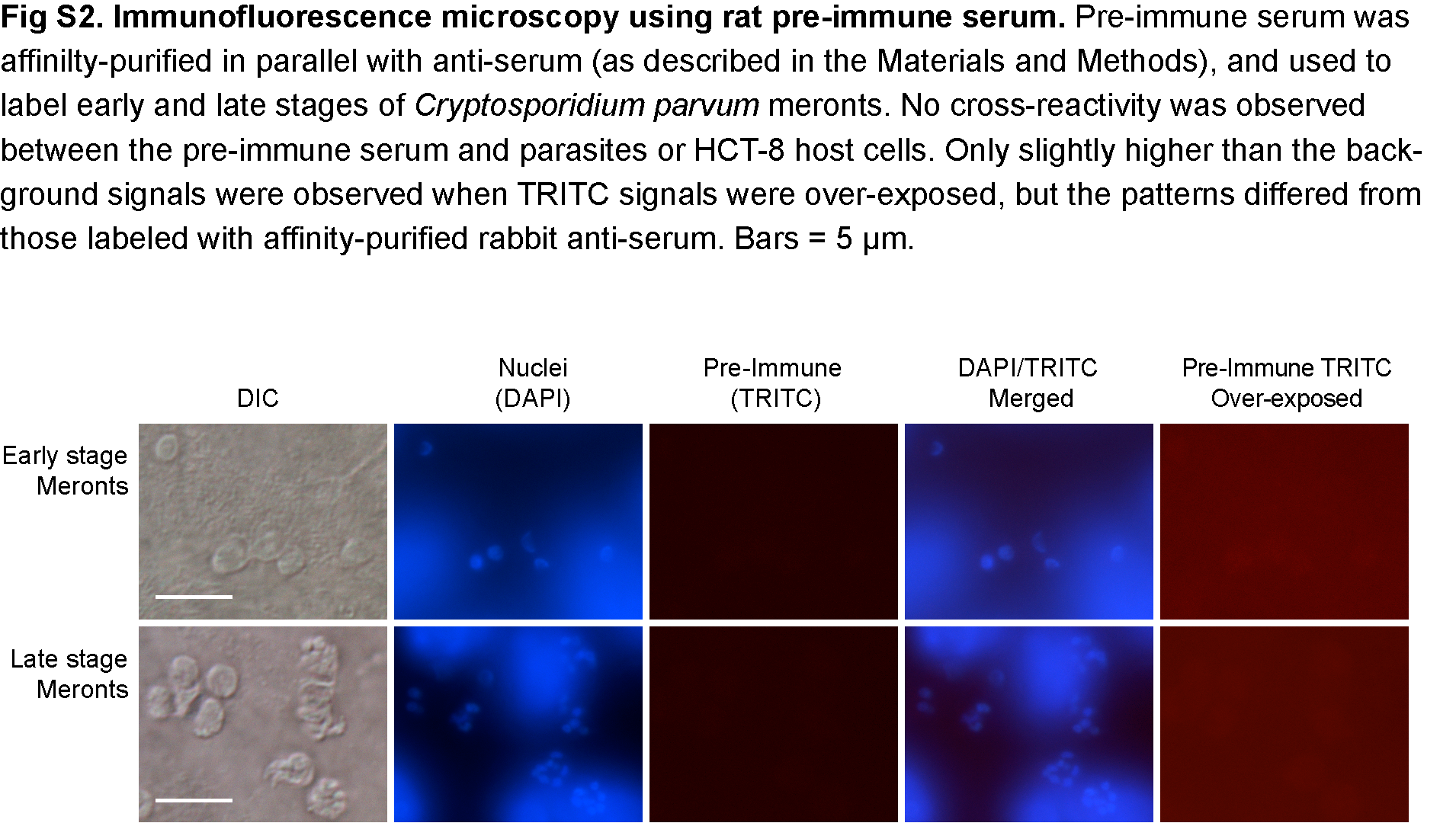

Supplement: S2 Fig — Pre-immune serum was affinilty-purified in parallel with anti-serum (as described in the Materials and Methods), and used to label early and late stages of Cryptosporidium parvum meronts. No cross-reactivity was observed between the pre-immune serum and parasites or HCT-8 host cells. Only slightly higher than the background signals were observed when TRITC signals were over-exposed, but the patterns differed from those labeled with affinity-purified rabbit anti-serum. Bars = 5 μm. (TIF) [file ppat.1005250.s002.tif]

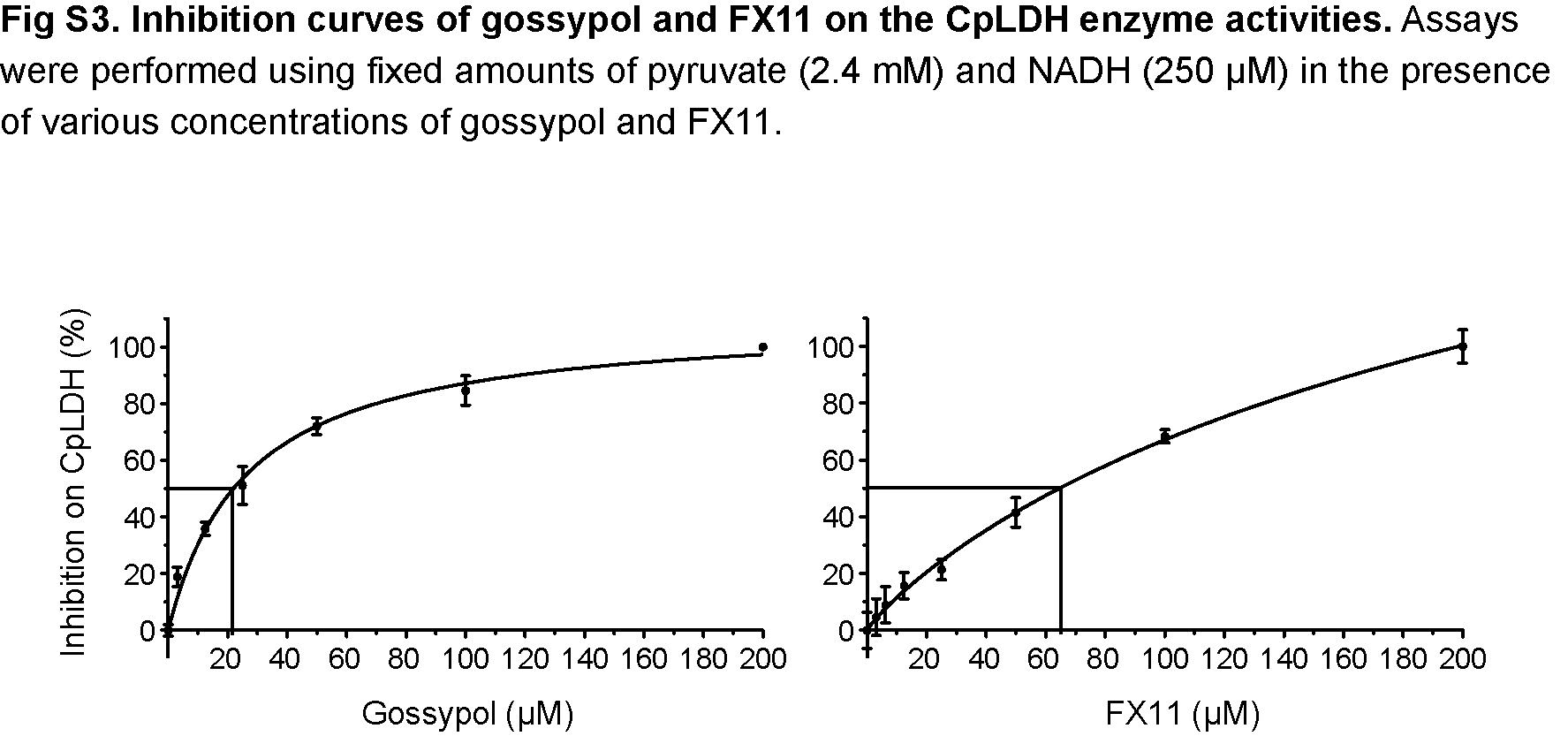

Supplement: S3 Fig — Assays were performed using fixed amounts of pyruvate (2.4 mM) and NADH (250 μM) in the presence of various concentrations of gossypol and FX11. (TIF) [file ppat.1005250.s003.tif]
